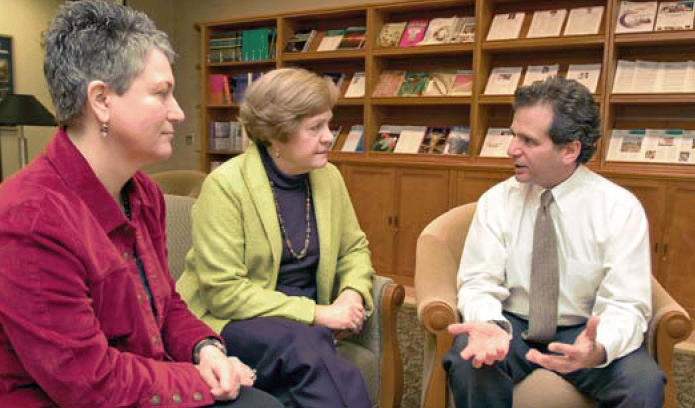# Environmental Health Sciences and the Community

**DOI:** 10.1289/ehp.114-1367852

**Published:** 2006-02

**Authors:** David A. Schwartz, Anne P. Sassaman, Gwen W. Collman

**Affiliations:** Director, NIEHS and NTP, E-mail: david.schwartz@niehs.nih.gov; Director, Division of Extramural Research and Training, Email: sassaman@niehs.nih.gov; Chief, Susceptibility and Population Health Branch, Email: collman@niehs.nih.gov

Environmental health science investigators train for many years to acquire the in-depth knowledge and expertise critical to successful research. The inherent components of hypothesis generation, study design, methodology, analysis, and interpretation of research which are central to the investigative process take years of dedicated work to develop. Moreover, the very aim of research—getting at the truth—requires an unbiased and impartial approach to answering the questions asked. However, scientists can’t always do patient-oriented research on their own, which is why the community is such an important component of our success at the NIEHS. Community partners can identify environmental exposures that are of concern, encourage the public to participate in research, help to set research priorities, and provide the bridge to developing and implementing effective interventions to reduce exposures and prevent disease.

The NIEHS and the broader community have had a distinguished and productive relationship. Under former director Ken Olden’s leadership, the NIEHS pioneered programs in community-based participatory research, health disparities, and environmental justice. Many research programs including the Core Centers, the Superfund Basic Research Program, the Centers for Children’s Environmental Health and Disease Prevention Research, the Breast Cancer and the Environment Research Centers, and the Centers for Population Health and Health Disparities each have active, vibrant community-based research or outreach activities that complement and extend the fundamental basic and disease-oriented research supported by the institute. The NIEHS has engaged the public by involving communities affected by environmental hazards in their neighborhoods in the research process. These programs have provided an ideal opportunity for communities to actively partner with scientists to engage in environmental health research, while also identifying the NIEHS as a leader and innovator in this novel approach to patient-oriented research.

The NIEHS has created direct channels of communication by holding regular Town Meetings throughout the United States, and by developing the Public Interest Liaison Group (PILG), made up of representatives of community, disease-advocacy, and environmental organizations, that meets regularly with the NIEHS leadership. The NIEHS also took the lead in establishing an Interagency Working Group on Community-Based Participatory Research involving a number of NIH institutes, the Environmental Protection Agency, the Department of Housing and Urban Development, and the Department of Transportation.

Some successful outcomes of the NIEHS and community partnerships include:

- public housing regulations to account for issues related to lead and allergen exposure;
- working with managed healthcare insurers to include asthma education services through home health aids to reduce reliance on hospital facilities in managing asthma symptoms among urban low-income, minority children;
- increased understanding and awareness of environmental triggers of asthma in schools and implementation of new cleaning protocols to reduce exposures;
- mobilization of local residents to raise concerns about pollution that resulted in citation of polluters in neighborhoods with high exposures;
- involvement of community advisory boards in planning and implementing family intervention studies to reduce asthma triggers and pesticide residue exposures to protect children’s health; and
- involvement of breast cancer survivor advocates as participants and key partners in facilitating the translation of research findings on the effects of early exposures on mammary gland development.

Strong partnerships between researchers and community members will remain critical to the success of the NIEHS in fulfilling our mission of understanding disease and improving public health.

While the institute’s community-based activities continue to change and evolve, the commitment that the NIEHS has to community-based research remains strong. As we prioritize areas for growth and development, the needs and health of communities are a specific focus of our vision. Our programs in integrative research, global environmental health, and exposure biology all focus on problems that evolve from and are relevant to the community. In addition, as part of our response to Hurricane Katrina, the NIEHS will develop a community-responsive research program to investigate the potential health effects of mold and microbial contamination of the home and work environment. Recognizing the importance of community input, we have also invited a PILG representative to regularly attend the NIEHS Advisory Council meetings.

As we move forward, community-based research programs will be integrated into the overall direction and goals of the NIEHS. We will stand by our prior commitments and ensure that we actively listen to our community stakeholders, involve them in critical research, and look to them for meaningful interventions. Strong partnerships between researchers and community members will remain critical to the success of the NIEHS in fulfilling our mission of understanding disease and improving human health.

## Figures and Tables

**Figure:**